# Arsenic and Intellectual Function: Bangladeshi Children at Risk

**Published:** 2004-09

**Authors:** John Tibbetts

In Bangladesh, naturally occurring arsenic contaminates some 10 million tube wells that about 30–40 million people depend on for drinking water. Scientists have already established that adults with heavy exposure to arsenic can suffer adverse impacts on cognitive functions such as learning and memory. However, there have been no well-controlled studies of the neurological consequences of arsenic exposure in children. This month, a group of U.S. and Bangladeshi researchers led by Gail Wasserman of Columbia University provides evidence that even modest exposure to arsenic in drinking water is associated with reduced intellectual function in children in Araihazar, Bangladesh **[*EHP* 112:1329–1333]**.

The investigators studied a group of 201 10-year-old children. The children’s parents were participating in an ongoing study of arsenic exposure among residents in a 25-square-kilometer region located about 30 kilometers east of Dhaka. The study site, Araihazar, was chosen because of its wide range of arsenic concentrations in drinking water.

The research team’s earlier survey of 6,000 contiguous tube wells in the region showed concentrations in individual wells ranging from less than 1 microgram per liter (μg/L) to 900 μg/L. Of the wells surveyed, 75% exceeded the World Health Organization (WHO) arsenic standard of 10 μg/L, and 53% exceeded the Bangladeshi standard of 50 μg/L.

In the current study, children and their mothers came to the research team’s field clinic for examination by a physician. The children provided urine specimens for the measurement of urinary arsenic and creatinine; about half also agreed to provide blood samples for measurement of blood lead and hemoglobin. Each child’s mother provided information about the family’s primary source of drinking water, and these sources were matched to the previously surveyed wells. In an effort to control for sociodemographic variables, the research team asked parents about parental age, education, and occupation, among other questions. The team also controlled for drinking water exposure to manganese, another known neurotoxicant (in their earlier survey, they had found that 82% of wells surveyed for manganese exceeded the WHO standard of 500 μg/L).

In addition to the medical evaluation, the children were assessed using an adaptation of the Wechsler Intelligence Scale for Children, version III (WISC-III). Because of the lack of standardized IQ measures in Bangladesh, Wasserman, a child psychologist, adapted the WISC-III for this cultural context. The WISC-III is a comprehensive series of tests that measures intellectual abilities such as comprehension and problem solving. Verbal subtests together provide a Verbal IQ, and a number of performance subtests (such as Picture Completion, Coding, Block Design, and Mazes) together provide a Performance IQ.

The researchers found that consumption of water contaminated by arsenic was associated with reduced intellectual function in a dose–response fashion. Children with exposures above 50 μg/L had significantly lower Performance and Full Scale scores than children with exposures under 5.5 μg/L. The children with the highest quartile of water arsenic also had marginally reduced Verbal scores. Lead and manganese exposures were not conclusively associated with impaired intellectual function, likely due to the low number of blood samples and confounding between arsenic and manganese, respectively.

The research team is working to curb exposure to arsenic in the study region. Since arriving in 2000, U.S. researchers, along with Bangladeshi colleagues, have overseen the installation of low-arsenic private and community wells and implemented a village education program that has successfully reduced some exposure. The authors note that the associations between arsenic and intellectual function were stronger for well-water concentrations than for urinary concentrations, which reflect recent exposure. The urinary concentrations at the time of testing may not reflect the full magnitude of the children’s earlier exposure, and the authors write that recently reduced exposure may explain the weaker associations between intellectual function and urine arsenic, compared to well-water arsenic.

